# Real-World Use of Dalbavancin in a United States Tertiary Referral Center

**DOI:** 10.7759/cureus.81505

**Published:** 2025-03-31

**Authors:** James Polega, Mudita Bhugra, Derek Vanderhorst, Jorgelina de Sanctis, Aaron Chang, Habiba Hassouna

**Affiliations:** 1 Infectious Disease, Corewell Health/Michigan State University, Grand Rapids, USA; 2 Pharmacy, Corewell Health/Michigan State University, Grand Rapids, USA; 3 Infectious Disease, Michigan State University College of Human Medicine, Grand Rapids, USA

**Keywords:** bacterial infection, dalbavancin, mrsa, outpatient parenteral antibiotic therapy, skin and soft tissue infection

## Abstract

Introduction

Outpatient management of serious bacterial infections can be difficult particularly in situations where outpatient parenteral antibiotic therapy may be difficult due to patient-specific factors such as history of medical non-compliance, unstable housing situations, and individuals who use injection drugs. Dalbavancin is a long-acting lipoglycopeptide antibiotic currently approved for the treatment of bacterial skin and soft tissue infections; however, it is often employed in the management of other infections caused by gram-positive organisms. Data regarding the off-label usage of dalbavancin remains an emerging area of study.

Methods

A retrospective cohort study of all 52 inpatients, ages 18-56, who received ≥1 dose of dalbavancin between November 2017 and February 2023 was conducted. Rates of treatment completion and clinical cure were assessed at 42 days post-dalbavancin treatment.

Results

Fifty-two adults were identified. Dalbavancin was used to treat skin/soft tissue infections in 12 patients (23.5%). Off-label uses accommodated for 40 patients, with diagnoses including as follows: bloodstream infection (21, 41.2%), osteomyelitis (12, 23.5%), septic arthritis (10, 13.3%), native valve infective endocarditis (six, 11.8%), prosthetic joint infection (three, 5.9%), epidural abscess (three, 5.9%), catheter-related bloodstream infection (one, 2%), and other infections (13, 25.5%). Among patients who completed therapy, cure, as assessed at day 42, was achieved in 35 (67.6%) patients. Intravenous (IV) drug and the need for facility placement to receive IV antibiotics use were the commonly cited reasons for dalbavancin utilization. Adverse events included mild elevation in serum aminotransferases which occurred in six patients (11.5%) and acute kidney injury which occurred in two (3.8%). There were no adverse events resulting in drug discontinuation.

Conclusions

Dalbavancin use, including off-label indications, such as bacteremia, septic arthritis, osteomyelitis, prosthetic joint infection, and others, appears safe and associated with favorable treatment responses. Therefore, it can be considered as an alternative treatment approach in patients who may not be candidates for traditional outpatient parenteral antimicrobial therapy.

## Introduction

Outpatient management of serious bacterial infections often requires agents with a degree of bioavailability and spectrum of activity that can be difficult to obtain without the use of intravenous (IV) antibiotics [1]. The administration of outpatient parenteral antibiotic therapy (OPAT) poses its own challenges such as the need for central catheter lines and associated complications, need for home nursing to assist with dose administration, and, in some cases, need for therapeutic drug monitoring [2,3]. These can pose challenges in properly executing OPAT in patients with a history of medical non-compliance and unstable housing situations and individuals who use injection drugs. Dalbavancin is a lipoglycopeptide antibiotic, first approved by the FDA in May 2014 for the treatment of acute bacterial skin and skin structure infections (ABSSSIs) due to *Staphylococcus aureus* (including methicillin-susceptible and methicillin-resistant strains), *Streptococcus pyogenes*, *Streptococcus agalactiae*, *Streptococcus dysgalactiae*, *Streptococcus anginosus* group, and *Enterococcus faecalis* (vancomycin-susceptible strains).

Due to its highly protein-bound nature, dalbavancin's terminal half-life is approximately 14 days. Trial data has demonstrated that bactericidal concentrations for *Staphylococcus aureus* are maintained for up to 14 days [4]. Following the introduction of dalbavancin, there has been significant interest in its use for other infections which require a prolonged course of therapy such as osteomyelitis and endocarditis, particularly in patients who are not candidates for traditional OPAT [5]. In 2015, Dunne et al. proposed that a two-dose regimen of dalbavancin administered as two 1,500 milligram doses given seven days apart should provide adequate drug concentration in bone and surrounding tissues for the treatment of osteomyelitis [6]. Subsequently, Rappo et al. conducted a randomized controlled trial of a two-dose regimen of 1,500 milligrams of dalbavancin administered seven days apart compared to the standard of care for bacterial osteomyelitis consisting of IV or oral (PO) antibiotics administered for 4-6 weeks. About 60% of pathogens in both study groups were *Staphylococcus aureus* with the majority being methicillin-susceptible isolates. Within the study group, 97% of the 70 patients treated with dalbavancin demonstrated clinical cure at six weeks of therapy compared to 88% of the group treated with standard-of-care antibiotics consisting of either vancomycin for the entire treatment duration or vancomycin for 5-16 days followed by transition to linezolid or levofloxacin [7].

A retrospective review conducted in Vienna, Austria, examined the use of dalbavancin in 27 patients with both native and prosthetic valve endocarditis due to gram-positive cocci. Nine of the infections in this group were due to *Staphylococcus aureus*. Most patients (88.9%) were treated with additional antibiotics prior to the initiation of dalbavancin. Antibiotic therapy prior to the initiation of dalbavancin varied across patients but generally consisted of a beta-lactam antibiotic combined with an additional agent such as daptomycin or rifampin. The authors reported a clinical cure in 25 of 27 patients [8]. Retrospective data from a multicenter study conducted in Spain demonstrated dalbavancin was not only an effective alternative to existing antimicrobial treatments for gram-positive infections but in many cases was cost-saving compared to other options [9]. A 2020 study evaluating the use of dalbavancin as a potential therapy for *Staphylococcus aureus* bacteremia and endocarditis in 18 patients, who were not otherwise candidates for traditional OPAT, demonstrated clinical cure rates of 44% leading the authors to conclude that dalbavancin may have a role as salvage or alternative therapy in individuals who are otherwise unable to utilize traditional OPAT. Patients included in the study population had received other antimicrobials, for an average duration of 12.3 days, prior to treatment with dalbavancin, the majority having received vancomycin [2]. Bresges et al. compiled three years of off-label dalbavancin treatment use in a recent paper across a variety of indications and reported a success rate of 75.4% from a cohort of 57 patients [10]. Wunsch et al. conducted a retrospective chart review of 101 patients treated with dalbavancin for various infectious indications across three medical centers, contributing additional real-world evidence of its efficacy. The study reported an overall clinical success rate of 89%, with cure rates exceeding 90% in cases of prosthetic joint infections and endocarditis [11]. In an effort to better understand the usage of dalbavancin at our center and to contribute to the broader body of evidence regarding its use, we have undertaken a retrospective review of dalbavancin usage at our institution since its introduction, in May of 2014. The specific aims of this study were as follows: (1) to better characterize the patient population receiving dalbavancin, including the indications for its use; (2) to assess the incidence and nature of any adverse effects associated with dalbavancin administration in this cohort; and (3) to evaluate treatment outcomes, with a focus on identifying common factors linked to treatment success or failure.

## Materials and methods

This is a retrospective chart review of individuals who received dalbavancin at our 1100-bed quaternary center, Corewell Health Butterworth and Blodgett Hospitals in Grand Rapids, Michigan, from November 2017 to February 2023 after obtaining approval from the Spectrum Health Institutional Review Board (approval number: 2021-466). Our pharmacy informatics group provided a list of all patients who received dalbavancin during the study period; we included all patients 18 years or older who received dalbavancin during the study period. Patients who had been planned to receive dalbavancin but missed their first dose were excluded; however, those who received their first dose but missed subsequent doses were included based on available data in the electronic health record. Additionally, any patient younger than 18 years old who received dalbavancin was excluded. Treatment outcomes were assessed at six-week and 12-week follow-up intervals to evaluate for cure of infection. Patient demographic factors, infection type, and causative organism were collected to help better understand the current use of dalbavancin at our institution. Medication-related adverse reactions including kidney injury, liver injury, and dermatologic reactions were collected. Mortality data, both all-cause mortality and mortality attributed to the patient's underlying infection, was obtained for the study population. All data was manually abstracted from our organization's electronic health record. Clinical cure was defined as the resolution of patients' initial infectious symptoms, with no concern for new or ongoing infectious processes by the treating clinician. Relapsed infection was defined as improvement in symptoms and/or laboratory parameters without the complete resolution of symptoms or complete normalization of laboratory parameters followed by subsequent worsening of clinical condition or laboratory parameters. Recurrent infection was defined as cessation of symptoms with the normalization of laboratory parameters followed by the development of symptoms or worsening in laboratory parameters. Relapse and recurrence were assessed at six-week follow-up visits after discharge from the hospital. Antimicrobial identification was performed using the matrix-assisted laser desorption/ionization-time-of-flight (MALDI-TOF) mass spectrometry, and antimicrobial susceptibility testing was performed via the use of a bioMérieux Vitek 2 instrument (Marcy-l'Étoile, France).

## Results

Patient characteristics

A total of 52 patients received treatment during our study period (22 female and 30 male). The median patient age was 49 (range 25-77). Age is presented as mean ± standard deviation. All other data points are presented as number (percentage). Demographics, patient comorbidities, and reasons for dalbavancin use are shown in Table 1.

**Table 1:** Patient demographics, comorbidities, and reasons for dalbavancin use

Demographics	
Age, mean ± SD	49 ± 17.5
Female, n (%)	22 (42.3)
Male, n (%)	30 (57.7)
Comorbidities	
Heart disease, n (%)	18 (40.9)
Liver disease, n (%)	13 (29.5)
Diabetes mellitus, n (%)	10 (22.7)
Chronic kidney disease, n (%)	8 (18.2)
Reasons for dalbavancin use	
Need for placement for IV antibiotic use, n (%)	20 (46.5)
Injection drug use, n (%)	20 (46.5)
Lack of adherence, n (%)	16 (37.2)
Side effect to conventional antibiotics, n (%)	2 (4.7)

Pathogens isolated/site of infection

The most common pathogens identified included methicillin-susceptible *Staphylococcus aureus* (MSSA) in 17 patients (37%), methicillin-resistant *Staphylococcus aureus* (MRSA) in 15 patients (32.6%), and coagulase-negative *Staphylococcus *in five patients (10.9%), including three isolates of *Staphylococcus epidermidis*, one isolate of *Staphylococcus lugdunensis*, one isolate of *Staphylococcus capitis*, and one isolate of *Staphylococcus hominis*.* Enterococcus faecalis* was isolated in three patients, and all isolates were vancomycin-susceptible (6.5%). *Streptococcus* species was isolated in two patients (4.3%): *Streptococcus agalactiae* in one patient and a Group C beta-hemolytic *Streptococcus* species in the other. Bacteremia was present in 21 patients (41.2%), skin and soft tissue infection was present in 12 patients (23.5%), osteomyelitis was present in 12 patients (23.5%), and native valve infective endocarditis was present in six patients (11.8%). Dalbavancin was not utilized in patients with organisms possessing a documented resistance to vancomycin. The site of infection and isolated pathogens are shown in Table 2.

**Table 2:** Infection type and pathogen isolated presented as number (%)

Infection type	
Bacteremia	21 (41.2)
Skin and soft tissue	12 (23.5)
Osteomyelitis	12 (23.5)
Septic arthritis	10 (19.6)
Native valve endocarditis	6 (11.8)
Prosthetic joint infection	3 (5.9)
Epidural abscess	3 (5.9)
Catheter-related bloodstream infection	1 (2)
Others	13 (25.5)
Pathogen isolated	
Methicillin-susceptible *Staphylococcus aureus*	17 (37)
Methicillin-resistant *Staphylococcus aureus*	15 (32.6)
Coagulase-negative *Staphylococcus*	5 (10.9)
*Enterococcus* species	3 (6.5)
*Streptococcus* species	2 (4.3)
Others	7 (15.2)

Adverse events

Elevated transaminases were noted in six patients (11.5%) during dalbavancin therapy, of whom three were receiving additional antimicrobial therapy including doxycycline, ciprofloxacin, cefadroxil, and daptomycin. Elevations in alkaline phosphatase were noted in two patients (3.8%) with both patients receiving concomitant doxycycline during the period in which abnormal labs were recorded. Acute kidney injury (AKI) occurred in two patients (3.8%), and neither of the patients who developed AKI were receiving concomitant antimicrobial therapy. Regarding the two patients who developed AKI, one had a history of type 2 diabetes with concomitant stage 3 chronic kidney disease, while the other had a prolonged ICU stay due to septic shock and experienced kidney injury during this hospitalization which had been improving until the administration of dalbavancin.

Dalbavancin indication/treatment outcomes

Reasons for dalbavancin use included concerns of the need for nursing facility placement for IV antibiotics in 20 patients (46.5%), concerns of injection drug use in 20 patients (46.5%), and medication non-compliance in 16 patients (37.2%). Several patients had multiple stated reasons for dalbavancin use such as IV drug use with concomitant medication non-compliance. The most used dosing strategy for dalbavancin was 1,500 mg on day 1 followed by 1,500 mg on day 8 which was used in 30 patients (57.7%). Forty-one patients (78.8%) completed their prescribed course of dalbavancin. A total of 35 of the 52 patients (67.6%) had a clinical cure at six weeks. Within the group that experienced clinical cure at six weeks, 33 of the 35 patients (94.3%) had completed their prescribed course of dalbavancin. The two patients who did not complete their prescribed course of therapy but experienced clinical cure had received an initial dose of 1,500 mg but did not receive their second dose. Both patients opted not to present to the infusion clinic for their second dose despite repeated attempts to contact them. Interestingly, neither patient was receiving concomitant antimicrobial therapy. Both of these individuals were being treated for MRSA bacteremia and had experienced blood culture clearance prior to the transition to dalbavancin.

Among those patients who experienced clinical cure, six patients were treated for bacteremia without obvious foci of infection. Five patients were treated for bacteremia with additional foci of infection, including discitis in two, osteomyelitis in one, ABSSSI in one, and suspected endocarditis with septic pulmonary emboli in one. Seven patients were treated for ABSSSI, and six patients were treated for osteomyelitis without evidence of metastatic infection.

Treatment for native valve endocarditis was attempted in six patients (five with MSSA and one with MRSA). Unfortunately, data to support clinical cure was only available in one patient, relapse occurred in one patient, recurrence of infection occurred in one patient, and three patients were lost to follow-up. The patient who achieved clinical cure was being treated for MRSA and did receive concomitant trimethoprim-sulfamethoxazole. MSSA was the cause of infection in the remaining five patients.

Among the patients who completed therapy but did not achieve a clinical cure, one was undergoing treatment for infective endocarditis, one for ABSSSI, one for septic arthritis with concomitant bacteremia, one for left ventricular assist device (LVAD) driveline infection, and one for septic arthritis without associated bacteremia. One patient who completed therapy for septic arthritis was lost to follow-up. Mortality during dalbavancin treatment occurred in one patient who was being treated for epidural abscess with concomitant bacteremia due to MSSA. The patient's death occurred two days after they had received their second infusion of dalbavancin as an outpatient. The patient's death was not communicated to the treating physician, and it is unclear based on available records if the death was directly attributable to infection. Characteristics of treatment failures are displayed in Table 3.

**Table 3:** Confirmed treatment failures LVAD: left ventricular assist device; MRSA: methicillin-resistant *Staphylococcus aureus*; MSSA: methicillin-susceptible *Staphylococcus aureus*

	Infection type/site	Pathogen	Dalbavancin dose	Antibiotic treatment prior to dalbavancin	Antibiotics administered with dalbavancin	Outcome at six weeks including mortality and infection status
Patient 1	Septic arthritis/bacteremia	MRSA	1,500 mg (day 1); 1,500 mg (day 7)	Vancomycin	Bactrim two double-strength tablets twice a day for 14 days	Patient: alive; infection: relapsed
Patient 2	Native valve endocarditis/bacteremia	MSSA	1,500 mg (day 1); 500 mg (day 7)	Cefazolin	None	Patient: alive; infection: relapsed
Patient 3	Skin and soft tissue	Unknown	1500 mg	Vancomycin and piperacillin-tazobactam	Doxycycline 100 mg twice a day	Patient: alive; infection: relapsed
Patient 4	Septic arthritis	MRSA	1000 mg (day 1); 500 mg (day 7)	Vancomycin	None	Patient: alive; infection: recurrent
Patient 5	LVAD driveline	*Enterococcus faecalis*, *Corynebacterium striatum*	1000 mg (day 1); 1000 mg (day 14)	Linezolid, vancomycin, doxycycline, and amoxicillin	None	Patient: alive; infection: relapsed

All patients treated with dalbavancin received antimicrobial therapy prior to the initiation of dalbavancin. The most utilized antibiotic prior to dalbavancin initiation was vancomycin in 36 patients (69.2%), followed by cephalosporin antibiotics in 34 patients (65.4%). Overall, a total of 35 patients (67.3%) in our study population had data available to support a cure of their infection. Among those who achieved clinical cure, 13 individuals (37% of treatment successes) received concomitant antimicrobial therapy. The most used concomitant antibiotic was doxycycline in six patients, followed by linezolid and trimethoprim-sulfamethoxazole. Multiple additional antibiotic strategies were utilized ranging from fully concomitant antibiotics during the duration of dalbavancin use to several weeks of antibiotics following dalbavancin treatment. Infection with *Staphylococcus aureus* was present in 18 individuals with nine being MSSA and nine being MRSA. In all cases of cure, dalbavancin was started after an initial course of therapy with another antimicrobial agent. 

## Discussion

Currently, the sole FDA indication for dalbavancin remains ABSSSIs due to gram-positive organisms [12]. However, a growing body of evidence points toward dalbavancin being a safe and effective treatment for more severe/deep-seated infections.

Our data are in line with previously reported cure rates for the use of dalbavancin for infections other than ABSSSI with an overall cure rate of 94.3% in individuals who completed prescribed courses of therapy [6-8]. Successful treatment was noted in multiple patients with osteomyelitis and ABSSSIs consistent with recent literature and the FDA-indicated use of dalbavancin. In patients who completed their course of dalbavancin therapy for bacteremia, successful treatment occurred in 10 of 16 (62.5%) cases; reported success rates in the literature for the treatment of bacteremia with dalbavancin range from 44% to 86% placing our results in line with existing data [2,11,13]. The number of patients treated for native valve endocarditis within our study was low with only six present. Unfortunately, only three patients with native valve endocarditis completed their course of therapy with only one demonstrating a cure of their infection. This demonstrates lower efficacy than what is typically reported in the literature [14].

From a safety perspective, dalbavancin appeared to be well tolerated with few adverse effects noted in our patients, and this is in line with what has previously been reported in the literature. A large meta-analysis including over 2,000 patients demonstrated that adverse events do not occur at a higher rate with dalbavancin compared to other antimicrobial agents [15]. No adverse effects in our study population resulted in the discontinuation of therapy, which is in line with previous studies that have found dalbavancin to be safe and well tolerated in most cases [16,17].

Within our patients who completed their prescribed course of dalbavancin and experienced treatment failure, there was an array of pathology including ABSSSI, septic arthritis, native valve endocarditis, and one LVAD driveline infection. From a microbiological standpoint, 80 of the failed cases were in patients whose infections were caused by *Staphylococcus aureus*; two patients had infections due to MSSA and two due to MRSA. The patient who failed treatment for ABSSSI had previously experienced multiple relapses of cellulitis and had been treated with multiple antibiotics previously. Underlying venous insufficiency, high body mass index, and onychomycosis are potential risk factors that might have contributed to their recurrent cellulitis, as opposed to failure of the drug. Both patients with septic arthritis who failed treatment had undergone appropriate source control procedures; one of the patients had a history of IV drug usage and comorbid ischemic heart disease, whereas the other patient had no significant medical comorbidities. The patient with LVAD driveline infection had ongoing concerns about inadequate source control which likely contributed to antimicrobial failure. However, given the difficulty in managing LVAD driveline infections, this outcome is not surprising [18]. It remains unclear what is responsible for the low rate of response in our native valve endocarditis population as our results appear to demonstrate worse efficacy than noted in larger series [16]. This may be due to the small sample size and because attempted treatment of MSSA with dalbavancin may have played a role as it is well known that anti-staphylococcal beta-lactams are superior to vancomycin in the treatment of MSSA [19]. Regarding the patients with endocarditis who completed therapy but failed, both were active IV drug users. Additionally, one of the two patients had chronic liver and kidney disease. Unfortunately, source control could not be obtained in either case of endocarditis as the patients were deemed not to be suitable surgical candidates by cardiothoracic surgery. In all cases of treatment failure except for one, the patient had been receiving vancomycin prior to the transition to dalbavancin.

Overall, given the small number of patients who experienced treatment failure despite completing treatment, it is difficult to generalize any patient characteristics which may have been related to treatment failures. From a limitations standpoint, the retrospective nature of our study limits any ability to determine causality. While selection bias can be a concern in retrospective work, we attempted to mitigate this by including all adult patients who received dalbavancin at our center. The single-center nature of the study may limit generalizability to other centers and practice settings. The size of the cohort limits the conclusions that can be drawn from the data given the small number of individuals treated for some conditions. Future work with larger retrospective cohorts or prospective studies focused on certain conditions would be useful to further define the role of dalbavancin.

## Conclusions

Our data reinforce previous studies which have demonstrated that dalbavancin can be used for indications outside ABSSSIs successfully, particularly when used as terminal therapy. Further work is needed to fully elicit the role of dalbavancin for the treatment of native valve endocarditis. Treatment failures for individuals who receive appropriate courses of dalbavancin appear to be rare.
